# MiR‐195 and miR‐497 suppress tumorigenesis in lung cancer by inhibiting SMURF2‐induced TGF‐β receptor I ubiquitination

**DOI:** 10.1002/1878-0261.12581

**Published:** 2019-11-08

**Authors:** Dong‐Kyu Chae, Jinyoung Park, Moonsoo Cho, Eunmi Ban, Mihue Jang, Young Sook Yoo, Eunice EunKyeong Kim, Ja‐Hyun Baik, Eun Joo Song

**Affiliations:** ^1^ Molecular Recognition Research Center Korea Institute of Science and Technology Seoul Korea; ^2^ School of Life Sciences and Biotechnology Korea University Seoul Korea; ^3^ Division of Bio‐Medical Science & Technology KIST School Korea University of Science and Technology Seoul Korea; ^4^ Biomedical Research Institute Korea Institute of Science and Technology Seoul Korea; ^5^ Graduate School of Pharmaceutical Sciences and College of Pharmacy Ewha Womans University Seoul Korea

**Keywords:** lung cancer, miR‐195, miR‐497, SMURF2, Transforming growth factor (TGF)‐β

## Abstract

SMURF2 is a member of the HECT family of E3 ubiquitin ligases that have important roles as a negative regulator of transforming growth factor‐β (TGF‐β) signaling through ubiquitin‐mediated degradation of TGF‐β receptor I. However, the regulatory mechanism of *SMURF2* is largely unknown. In this study, we identified that micro(mi)R‐195 and miR‐497 putatively target *SMURF2* using several target prediction databases. Both miR‐195 and miR‐497 bind to the 3′‐UTR of the *SMURF2* mRNA and inhibit *SMURF2* expression. Furthermore, miR‐195 and miR‐497 regulate SMURF2‐dependent TβRI ubiquitination and cause the activation of the TGF‐β signaling pathway in lung cancer cells. Upregulation of miR‐195 and miR‐497 significantly reduced cell viability and colony formation through the activation of TGF‐β signaling. Interestingly, miR‐195 and miR‐497 also reduced the invasion ability of lung cancer cells when cells were treated with TGF‐β1. Subsequent *in vivo* studies in xenograft nude mice model revealed that miR‐195 and miR‐497 repress tumor growth. These findings demonstrate that miR‐195 and miR‐497 act as a tumor suppressor by suppressing ubiquitination‐mediated degradation of TGF‐β receptors through SMURF2, and suggest that miR‐195 and miR‐497 are potential therapeutic targets for lung cancer.

## Graphical abstract

We demonstrated that ectopic expression of miR‐195 and miR‐497 by mimics suppressed TβR1 ubiquitination and degradation through the repression of SMURF2. These results suggest that the reduction of SMURF2 gene expression by miR‐195 or miR‐497, which results in activation of transforming growth factor‐β signaling, has an important role in inhibiting lung cancer.

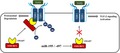

**Abbreviations**
GEO: Gene Expression Omnibus
miRNA: microRNA
NSCLC: non‐small‐cell lung cancer
PMSF: phenylmethylsulfonyl fluoride
qRT‐PCR: quantitative real‐time PCR
SBE: Smad‐binding element
SMURF2: SMAD‐specific E3 ubiquitin protein ligase 2
TGF‐β: transforming growth factor‐β
TβRI: TGF‐β receptor I
TβRII: TGF‐β receptor II

## 1 Introduction

Transforming growth factor‐β (TGF‐β) signaling acts as a key regulator of tissue homeostasis and carcinogenesis, according to growing clinical evidence. Dysregulation of the TGF‐β signaling pathway correlates with crucial roles in tumor initiation development and metastasis (Colak and Ten Dijke, 2017). The roles of the TGF‐β signaling pathway depend on the different stages of cancer and on the differences between genetic backgrounds (Zarzynska, 2014). In early‐stage cancer cells, this signaling pathway has tumor‐suppressor functions including apoptosis and inhibition of cell proliferation. However, its high activity in late‐stage cancer tends to promote cell growth and to exhibit aggressive behavior. Thus, understanding the complex roles of TGF‐β signaling is important to apply the appropriate cancer treatment.

Transforming growth factor‐β activation is initiated by assembling a receptor complex with TGF‐β receptor types I and types II (TβRI and TβRII) by binding ligands (Ikushima and Miyazono, 2010). The phosphorylated regulatory SMAD (R‐SMAD) proteins by activated TβRI can form a heteromeric complex with the common signaling transducer SMAD4; and then translocate into the nucleus. Activated R‐SMAD‐SMAD4 complexes bind other DNA‐binding transcription factors for the transcription of hundreds of TGF‐β target genes (Shi and Massagué, 2003). The sensitivity and duration of TGF‐β signaling are negatively regulated by SMAD‐specific E3 ubiquitin protein ligase 2 (SMURF2) and inhibitory SMAD. The SMURF2 and SMAD7 complex can bind to the activated TGF‐β receptor complex and then prevent the access of R‐SMADs to the TGF‐β receptor complex. This complex leads to ubiquitination and proteasomal degradation of the TGF‐β receptor complex and thus attenuates the TGF‐β signaling pathway (Kavsak *et al.*, 2000; Yan *et al.*, 2009).

SMURF2 has been reported to have a dual role in cancer, acting as both a tumor promoter and a tumor suppressor. Recent studies have reported that SMURF2 suppresses tumors by controlling the epigenetic landscape of the histone modification through RNF20 (Blank *et al.*, 2012) and also decreases the TGF‐β‐induced EMT in NMuMG cells (Chandhoke *et al.*, 2016). Conversely, induction of SMURF2 enhances tumor metastasis in a nude mouse model and increases invasion and migration of breast cancer cells (Jin *et al.*, 2009) and inhibits cell apoptosis by promoting p53 degradation by stabilizing the E3 ligase MDM2 (Nie *et al.*, 2010). In addition, SMURF2 also has a significant role in ubiquitin‐mediated proteasomal degradation of TGF‐β signaling components such as SMAD1, SMAD2 (Lin *et al.*, 2000; Zhang *et al.*, 2001), SMAD3 (Wu *et al.*, 2008) and TβRI (Kavsak *et al.*, 2000).

MiR (miRNA) are small non‐coding RNA molecules involved in the regulation of gene expression through base‐pairing with complementary sequences of the 3′‐UTR of target mRNA. They are associated with numerous cancer‐relevant biological processes such as cell proliferation, differentiation, cell death and motility (Hernández *et al.*, 2018; Macfarlane and Murphy, 2010). MiR‐195 and miR‐497 have been reported to inhibit cell cycle in HCC by directly binding to *CCNE1*, *CDC25A*, *CDK4* and *CDK6* (Furuta *et al.*, 2013) and suppress breast cancer cell proliferation and invasion by regulating *Raf‐1* and *CCND1* (Li *et al.*, 2011). In addition, miR‐195 and miR‐497 have been reported to regulate TGF‐β signaling by inhibiting the components of TGF‐β signaling such as *SMAD2*, *SMAD4* and *SMAD7* (Duan and Chen, 2016; Hu *et al.*, 2016; Jafarzadeh *et al.*, 2016; Liu *et al.*, 2016). Furthermore, miR‐195 and miR‐497 expression were downregulated and they act as tumor suppressors in various tumors including non‐small‐cell lung cancer (NSCLC), cervical cancer and breast cancer (Li *et al.*, 2011; Su *et al.*, 2016; Wang *et al.*, 2010; Zhao *et al.*, 2013). However, the functions of miR‐195 and miR‐497 have not been fully elucidated.

The current study demonstrated that miR‐195 and miR‐497 negatively regulate *SMURF2* expression and SMURF2‐dependent TβRI ubiquitination, thereby increasing the activation of TGF‐β signaling. In addition, miR‐195 and miR‐497 have inhibited cancer cell growth, colony formation, and invasion in cancer cell lines and attenuated tumorigenesis in a xenograft mouse model. This study provides further insight into the novel molecular mechanism underlying lung tumorigenesis and may help to develop a new prognosis marker or therapeutic target for lung cancer.

## 2 Materials and methods

### 2.1 Cell culture

L132 (non‐transformed lung epithelial cell), A549, H157, H1299, H1703 (lung cancer cell lines) and HEK293T cells were purchased from Korean Cell Line Bank (Seoul, Korea). All lung cancer cell lines and L132 cells were maintained in RPMI‐1640 medium supplemented with 10% FBS and 1% penicillin‐streptomycin (Hyclone, Logan, UT, USA). HEK293T cells were maintained in a DMEM medium supplemented with 10% FBS and 1% penicillin‐streptomycin in a humidified chamber with 5% CO_2_ at 37 °C.

### 2.2 Transfections of miRNA

The miRNA inhibitors and mimics of miR‐NC, miR‐195 and miR‐497 were purchased from Genolution (Seoul, Korea). Upon reaching 60–70% confluence, the A549 cells were transfected with 50 nm of inhibitors or mimics of miR‐NC, miR‐195 and miR‐497 using Lipofectamine 2000 (Invitrogen, Carlsbad, CA, USA) according to the manufacturer’s instructions. The expression levels of miR‐195 and miR‐497 were quantified 48 h after transfection and the cells were used for a western blot analysis.

### 2.3 Construction of luciferase reporter plasmid and luciferase reporter assays

The putative miR‐195 and miR‐497 target sequences of *SMURF2* mRNA were determined using TargetScan, miRanda and miRWalk. The *SMURF2* 3′‐UTR of the miR‐195 and miR‐497 was directly synthesized (Cosmogenetech, Seoul, Korea) and cloned into pmirGLO (Promega Corp., Madison, WI, USA) between the *Not*I and *Xba*I multi‐cloning sites. Inserts were ligated into the pmirGLO vector and transformed into DH5α‐competent cells. Target sequences of *SMURF2* for miR‐195 and miR‐497 were as follows: 3′‐UTR_WT 5′‐AAA CTA GCG GCC GCT AGT ATG AGG CCA CAT TCA GCT GCT ATT TAA T‐3′ and 5′‐CTA GAT TAA ATA GCA GCT GAA TGT GGC CTC ATA CTA GCG GCC GCT AGT TT‐3′; 3′‐UTR_mut 5′‐AAA CTA GCG GCC GCT AGT ATG AGG ACC CCT TCA TCG GAT ATT TAA T‐3′ and 5′‐CTA GAT TAA ATA TCC GAT GAA GGG GTC CTC ATA CTA GCG GCC GCT AGT TT‐3′. Dual‐luciferase assay was performed by co‐transfecting A549 cells with 200 ng pmirGLO luciferase reporter vector containing wild‐type or mutant 3′‐UTR of *SMURF2* and 25–100 nm miR‐195 and miR‐497 mimics using Lipofectamine 2000 Transfection Reagent (Invitrogen).

To measure downstream TGF‐β signaling activity in response to TGF‐β1, A549 cells were co‐transfected with 100 ng of Smad‐binding element (SBE)‐luciferase reporter from the Cignal SMAD Reporter Assay Kit (Qiagen, Germantown, MD, USA) and 50 nm mimics of miR‐195 or miR‐497 for 24 h. The cells were treated with 5 ng·mL^−1^ TGF‐β1 for 24 h. Luciferase activity was measured using a Dual‐Luciferase Reporter Assay Kit (Promega Corp.), and firefly luciferase activity was normalized to *Renilla* luciferase activity.

### 2.4 Western blot analysis

Cells were lysed using protein extraction buffer at 48 h after transfection, and protein concentration was quantitated by BCA protein assay. Lysates (30 µg) were resolved by SDS/PAGE and transferred onto nitrocellulose membrane (Pall corporation, Port Washington, NY, USA). The following antibodies were purchased from Cell Signaling Technology (Danvers, MA, USA): SMAD2 (catalogue no. 5339); phospho‐SMAD2 (catalogue no. 3108); SMAD3 (catalogue no. 9523); p21 (catalogue no. 2947); Santa Cruz Biotechnology (Delaware, CA, USA): SMURF2 (catalogue no. sc‐25511); Na^+^/K^+^ ATPase α (catalogue no. sc‐48345), (AbFrontier, Seoul, Korea): anti‐β‐actin (catalogue no. LFPA0207), (Sigma‐Aldrich, St. Louis, MO, USA): anti‐HA (catalogue no. H6908); anti‐Flag (catalogue no. F1804), (Abcam, Cambridge, UK): anti‐TβRI (catalogue no. ab31013); phospho‐SMAD3 (catalogue no. ab52903). Western Blotting Luminol Reagent (Santa Cruz Biotechnology, Santa Cruz, CA, USA) was used to detect protein according to the manufacturer’s instructions. The membranes were visualized with an ATTO image analyzer (ATTO, Tokyo, Japan).

### 2.5 Ni‐NTA pulldown assay

To assess ubiquitination, the pCMV‐HA‐TβRI, pCMV‐His‐Ub and pCMV5B‐Flag‐SMURF2‐WT expression vectors were co‐transfected with mimics or inhibitors of miR‐NC, miR‐195 and miR‐497 using the calcium chloride transfection method. The pCMV‐HA‐TβRI was a kind gift from K.‐M. Yang (Seoul National University) and pCMV5B‐Flag‐SMURF2‐WT was a gift from J. Wrana (Addgene plasmid # 11746) (Kavsak *et al.*, 2000). The cells were treated with 10 μm MG132 prior to harvesting to inhibit proteasomal degradation.

Transfected HEK293T cells were harvested with urea lysis buffer (8 m urea, 10 mm imidazole, 300 mm NaCl, 50 mm Na2HPO4, 50 mm Tris and 1 mm PMSF, pH 8.0). Cell lysates were lysed by sonication and mixed with Ni‐NTA agarose bead (Qiagen) for 4 h at 4 °C in a rotator. The agarose was washed five times with urea lysis buffer (8 m urea, 20 mm imidazole, 300 mm NaCl, 50 mm Na_2_HPO_4_, 50 mm Tris and 1 mm PMSF, pH 8.0). The bound protein was eluted off the Ni‐NTA agarose bead in 2× SDS sample buffer by boiling at 95 °C. Affinity purified protein samples were analyzed by SDS/PAGE.

### 2.6 Isolation of cell surface protein

A549 cells were transfected with inhibitors or mimics of miR‐NC, miR‐195 or miR‐497 together with pCMV‐HA‐TβRI, pCMV5B‐Flag‐SMURF2‐WT, or pCMV5B‐Flag‐SMURF2^C716A^ expression vector. Cell surface biotinylation in A549 cells was carried out with Pierce™ Cell Surface Protein Isolation Kit (Thermo Fisher, Rockford, IL, USA) according to the manufacturer’s protocol. Cell surface protein was labeled with EZ‐LINK Sulfo‐NHS‐SS‐biotin for 30 min at 4 °C. Excess biotin was quenched with quenching solution. Cells were treated with lysis buffer and centrifuged at 10 000 ***g*** for 2 min at 4 °C. Supernatant was reacted with immobilized NeutrAvidin agarose slurry in column and eluted by the sample buffer containing DTT. Surface proteins were resolved and analyzed on a SDS/PAGE.

### 2.7 Quantitative real‐time PCR

Total RNA was extracted from cells using Trizol Reagent (Ambion, Carlsbad, CA, USA) according to the manufacturer’s instructions. To analyze miR‐195 and miR‐497 expression, cDNA was synthesized by a Mir‐X miRNA First‐Strand Synthesis Kit (Takara, Dalian, China). The primers used to amplify miR‐195 and miR‐497 were the forward 5′‐UAG CAG CAC AGA AAU AUU GGC‐3′, 5′‐CAG CAG CAC ACU GUG GUU UGU‐3′ and a universal reverse primer from the Mir‐X miRNA qRT‐PCR SYBR Kit (TOYOBO, Osaka, Japan). Forward and reverse primers of U6 small nuclear RNA were provided by the Mir‐X miRNA qRT‐PCR SYBR Kit.

To detect *SMURF2* and *CDKN1A* mRNA, cDNA was synthesized by a ReverTra Ace qPCR RT Kit (TOYOBO). The *SMURF2* primers were F‐5′‐TTG GCT CTG CAG AAA GGA TT‐3′ and R‐5′‐CCA CAG CTT TCC AGA ACC AT‐3′. The *CDKN1A* primers were F‐5′‐ CAC CAC TGG AGG GTG ACT TC‐3′ and R‐5′‐ATC TGT CAT GCT GGT CTG CC‐3′. The β‐actin primers were F‐5′‐CTC TTC CAG CCT TCC TTC CT‐3′ and R‐5′‐AGC ACT GTG TTG GCG TAC AG‐3′. The quantitative real‐time PCR (qRT‐PCR) was performed using THUNDERBIRD SYBR qPCR Mix (TOYOBO). The miRNA and mRNA expressions were normalized to those of U6 small nuclear RNA and β‐actin, respectively.

### 2.8 Cell proliferation assay

A549 cells were seeded into 96‐well plates (4000 cells per well) at RPMI containing 10% FBS per well. After 24 h, the cells were transfected with 10 nm of mimics or inhibitors of miR‐NC, miR‐195 or miR‐497 alone, or together with flag‐SMURF2. WST‐1 assays (Dogen, Seoul, Korea) were conducted to measure cell proliferation at different time points. A 10‐μL aliquot of WST‐1 reagent was mixed with 100 µL culture medium and incubated for 30 min in a cell incubator. The absorbance was subsequently measured on a Microplate Absorbance Reader (Bio‐Rad, Hercules, CA, USA) using a test wavelength of 450 nm.

### 2.9 Invasion assay

Cell invasion assays were performed in 6.5‐mm Transwells (8.0 μm pore size) (Coring Incorporation, New York, NY, USA). Transwell invasion chamber containing polycarbonate filters were coated with diluted Matrigel (BD Bioscience, Billerica, MA, USA). Cells at a concentration of 1 × 10^5^ were re‐suspended in 200 μL of serum‐free RPMI and plated in the upper chamber with a Matrigel‐coated membrane. The lower chamber was filled with 700 μL RPMI medium containing 10% FBS and 5 μg·mL^−1^ fibronectin. After 48 h, the non‐invading cells on the top surface of the filter were manually removed by wiping with a cotton swab. Invading cells were fixed in methanol and stained with Giemsa for 15 min. The number of cells was counted under a magnification of ×20.

### 2.10 Colony formation

Cells were transfected with 10 nm of miR‐NC, miR‐195 or miR‐27a, or anti‐NC, anti‐miR‐195 or miR‐497 in 60‐mm culture dishes. After 48 h, 500 cells were transferred to 35‐mm culture plates. After incubation for 10 days, cells were rinsed with PBS and fixed with 4% formaldehyde for 1 h. Fixed cells were stained with Crystal violet.

### 2.11 A549 tumor xenografts

All the animal care and experiments were followed according to the regulation of the Institutional Animal Care and Use Committee in Korea Institute of Science and Technology (KIST). To generate A549 tumor‐bearing xenografts, A549 cells at a concentration of 1 × 10^7^ mixed with 50% Matrigel (BD Biosciences) were implanted subcutaneously in the flanks of female nude mice. When the tumor volume reached approximately 100 mm^3^, different miRNA including miR‐NC, miR‐195, and miR‐497, were administrated intratumorally at a dose of 4 mg·kg^−1^. The treatment of miRNA in A549 xenografts was performed with an interval of 3 days for 2 weeks, and *in vivo* anti‐cancer activity was evaluated by monitoring each tumor volumes every 3 days.

### 2.12 Tissue immunofluorescence staining and TUNEL assay

To detect expression of SMURF2 in the excised tumor sections, immunofluorescence staining was conducted according to standard procedures. To obtain tumor sections, all tissues were embedded in Optimal Cutting Technique (OCT) compound (Leica, Bensheim, Germany), and immunofluorescence staining was conducted using primary antibody against SMURF2 (Santa Cruz; 1 : 100), followed by Alexa Fluor 549‐conjugated goat anti‐rabbit IgG (Thermo Fisher; 1 : 500).

To access the induction of apoptosis *in vivo*, TUNEL staining was conducted in the fixed tumor sections using an *in situ* cell death detection kit (Roche, Mannheim, Germany) according to the manufacturer’s instructions. To visualize the nucleus, the tumor sections were mounted with DAPI mounting medium (Vector Laboratories, Burlingame, CA, USA) and evaluated using a ZEISS LSM 700 confocal microscope (Oberkochen, Germany).

### 2.13 Statistical analyses

All data were expressed as mean ± SD, except where indicated as ± SEM in the legends, of at least three independent experiments. Two‐tailed *t*‐tests, one‐way and two‐way ANOVA were performed using graphpad™ 4.0 software (SanDiego, CA, USA). Statistical significance was determined at *P* < 0.05.

## 3 Results

### 3.1 The expression of miR‐195 and miR‐497 in lung cancer patients

**Figure 1:**
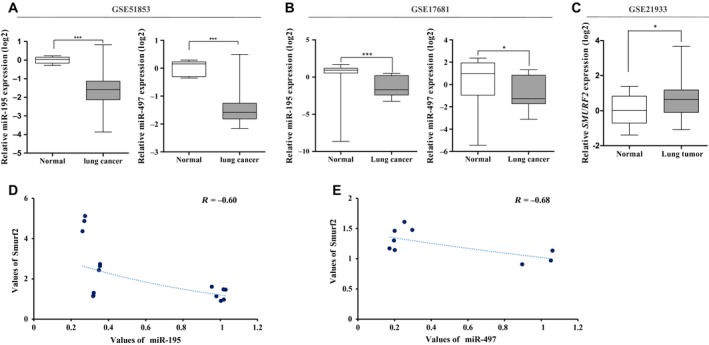
MiR‐195 and miR‐497 are downregulated in lung cancer patients and lung cancer cell lines. (A) MiR‐195 and miR‐497 expression levels were evaluated in normal tissue and NSCLC in the http://www.ncbi.nlm.nih.gov/geo/query/acc.cgi?acc=GSE51853 dataset. (B) Relative miR‐195 and miR‐497 levels from total blood of lung cancer and control samples in http://www.ncbi.nlm.nih.gov/geo/query/acc.cgi?acc=GSE17681. (C) Differential expression of *SMURF2* genes was obtained from the mRNA microarray data of the http://www.ncbi.nlm.nih.gov/geo/query/acc.cgi?acc=GSE21933 dataset. The miRNA expression values were transformed to log2 (tumor/normal). The miRNA expression levels are shown on the *y*‐axis (log2 intensity: *P ≤ 0.05, ***P ≤ 0.001, Student’s *t*‐test). (D,E) Correlation between the levels of miR‐195 or miR‐497 and *SMURF2* gene in lung cancer cell lines. *R* values were obtained from using Pearson’s correlation.

MiR‐195 and miR‐497 in the same cluster are located within the same chromosomal region (17p13.1) and simultaneously transcribed by a single RNA polymerase. Previous studies have shown that this region is deleted in peritoneal carcinoma and breast cancer, and the expressions of miR‐195 and miR‐497 are downregulated in HCC, breast cancer and adrenocortical carcinoma (Flavin *et al.*, 2009; Furuta *et al.*, 2013; Koduru *et al.*, 2017; Li *et al.*, 2011). As tumor suppressors, these miRNA have an important role in regulating cell proliferation, apoptosis and metastasis (Gu *et al.*, 2016; Guo *et al.*, 2014; Han *et al.*, 2015; Wang *et al.*, 2014; Zhu *et al.*, 2012). Furthermore, miR‐195 and miR‐497 could be considered potential prognostic biomarkers in NSCLC, gastric cancer, osteosarcoma, renal cell carcinoma, pancreatic cancer and breast cancer (Igglezou *et al.*, 2014; Shen *et al.*, 2016; Su *et al.*, 2016; Xu *et al.*, 2014; Zhao *et al.*, 2013). However, the functions of miR‐195 and miR‐497 as well as their target genes have not yet been fully elucidated in lung cancer. Therefore, we first investigated miR‐195 and miR‐497 expression in a large cohort of lung cancer tissues (126 non‐small‐cell lung cancer specimens and five matched normal lung samples) and blood (17 lung cancer and 19 control samples) available from the Gene Expression Omnibus (GEO) database (accession number http://www.ncbi.nlm.nih.gov/geo/query/acc.cgi?acc=GSE51853 and http://www.ncbi.nlm.nih.gov/geo/query/acc.cgi?acc=GSE17681) of the National Center for Biotechnology Information (NCBI). We found that miR‐195 and miR‐497 expression levels were significantly downregulated in the tissue (Fig. 1A) and blood samples (Fig. 1B) of lung cancer patients.

To study the functional mechanism of miR‐195 and miR‐497, we searched for candidate target genes of miR‐195 and miR‐497. Using target prediction databases such as TargetScan, miRanda and miRWalk 2.0, we found that the 3′‐UTR of the *SMURF2* mRNA has binding sequences for miR‐195 and miR‐497. Analysis of the *SMURF2* gene expression in the http://www.ncbi.nlm.nih.gov/geo/query/acc.cgi?acc=GSE21933 dataset (gene expression profiles of tumor and paired normal lung tissues from primary NSCLC patients in Taiwan) from the GEO database showed that *SMURF2* expression was upregulated in NSCLC (Fig. 1C). In addition, we examined miR‐195/497 and *SMURF2* expression levels in lung cancer cell lines. As those of lung cancer tissue and blood samples, miR‐195 and miR‐497 expressions were lower, whereas *SMURF2* expression was higher in most lung cancer cell lines than in the controls (Fig. S1A,B). These data seem to have a tendency for a negative correlation between the abundance levels of miR‐195 or miR‐497 and the mRNA levels of *SMURF2* (Fig. 1D,E). Taken together, these data suggest that the low miR‐195 and miR‐497 expressions and high *SMURF2* expression are associated with the biological process of tumorigenesis in lung cancer patients.

### 3.2 MiR‐195 and miR‐497 regulate the *SMURF2* gene by directly targeting with *SMURF2* 3′‐UTR

**Figure 2:**
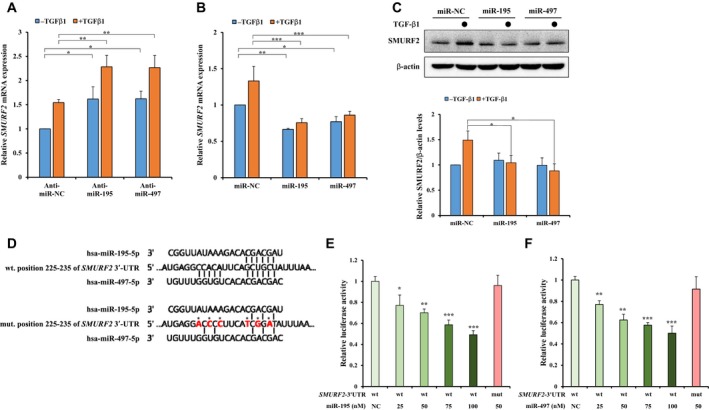
*SMURF2* is a direct target of miR‐195 and miR‐497. (A, B) The effects of miR‐195 and miR‐497 on *SMURF2* gene expression in A549 cells. The cells were transfected with the inhibitor for miR‐NC, miR‐195 or miR‐497 or with the mimic for miR‐NC, miR‐195 or miR‐497 for 48 h and then treated with or without 5 ng·mL^−1^ TGF‐β1 for 4 h, and *SMURF2* gene expression determined by qRT‐PCR. A two‐way ANOVA with Bonferroni posttest was used for statistical analysis. All data represent mean ± SEM: *P ≤ 0.05, **P ≤ 0.01, ***P ≤ 0.001. (C) Western blot analysis was performed to measure the changes in the SMURF2 protein expression. A549 cells were transfected with the mimic of miR‐NC, miR‐195 or miR‐497 for 48 h and then treated with or without 5 ng·mL^−1^ TGF‐β1 for 24 h. Cell lysates were immunoblotted with anti‐SMURF2, and β‐actin was used as a loading control (top). Quantification of SMURF2 levels was done considering the amount of β‐actin protein in each case (bottom). A one‐way ANOVA with Dunnett’s multiple comparison test was used for statistical analysis: *P ≤ 0.05 versus control. (D) Putative miR‐195 and miR‐497 binding site in the human *SMURF2* 3′‐UTR and luciferase constructs with the wild‐type and mutant miR‐195 and miR‐497 target sequences. The red colors indicate the mutant sequences of the 3′‐UTR. (E,F) A549 cells were co‐transfected with 25–100 nm of the miR‐195 or miR‐497 mimic together with the pmirGLO dual‐luciferase vectors containing the wild type or mutant 3′‐UTR of *SMURF2*. Data are represented as the mean ratios of Renilla to Firefly luciferase activity and are normalized relative to the negative control. A one‐way ANOVA with Dunnett’s multiple comparison test was used for statistical analysis: *P ≤ 0.05, **P ≤ 0.01, ***P ≤ 0.001 versus control.

To verify our hypothesis that miR‐195 and miR‐497 might regulate the *SMURF2* mRNA, we examined the *SMURF2* mRNA and protein expression after transfection with mimics or inhibitors of miR‐195 and miR‐497. We first confirmed the transfection efficiency of miR‐195 and miR‐497 using qRT‐PCR. As shown in Fig. S2A,B, the expression of miR‐195 and miR‐497 were upregulated or downregulated by transfection with its corresponding mimics or inhibitors, respectively. Next, we examined whether miR‐195 and miR‐497 regulate the *SMURF2* mRNA and protein expression in A549 cells. The inhibitors of miR‐195 and miR‐497 upregulated the expression of the *SMURF2* gene relative to the negative control (Fig. 2A), whereas the overexpression of miR‐195 and miR‐497 by the mimics significantly downregulated the *SMURF2* mRNA (Fig. 2B). In A549 cells treated with TGF‐β1, *SMURF2* mRNA expression was induced by TGF‐β1, and the inhibitors of miR‐195 and miR‐497 enhanced the *SMURF2* mRNA expression more than the control, whereas we observed that the miR‐195 and miR‐497 mimics downregulated the TGF‐β1‐induced increase of the *SMURF2* expression (Fig. 2A,B). Unfortunately, we observed that the effects of the inhibitors of miR‐195 and miR‐497 were insignificant on the TGF‐β1‐induced SMURF2 protein expression (data not shown). However, the overexpression of miR‐195 and miR‐497 by the mimics reduced TGF‐β1‐induced SMURF2 protein (Fig. 2C).

Bioinformatics databases predicted a potential miR‐195 and miR‐497 binding site in the 3′‐UTR of *SMURF2*. Based on these data, we cloned the wild‐type and the mutant miR‐195 and miR‐497 binding sequences of the *SMURF2* 3′‐UTRs. Using the pmirGLO dual‐luciferase reporter assay, we confirmed that miR‐195 and miR‐497 directly target the 3′‐UTR of the *SMURF2* gene (Fig. 2D). Additionally, 25–100 nm of the miR‐195 and miR‐497 mimics led to a significant decrease in luciferase activity for the wild‐type 3′‐UTR reporter gene in a dose‐dependent manner but failed to inhibit the activity of the mutant 3′‐UTR reporter gene (Fig. 2E,F). These findings indicate that miR‐195 and miR‐497 directly regulate the *SMURF2* gene expression through post‐transcriptional repression.

### 3.3 MiR‐195 and miR‐497 regulate the ubiquitination of TβRI

**Figure 3:**
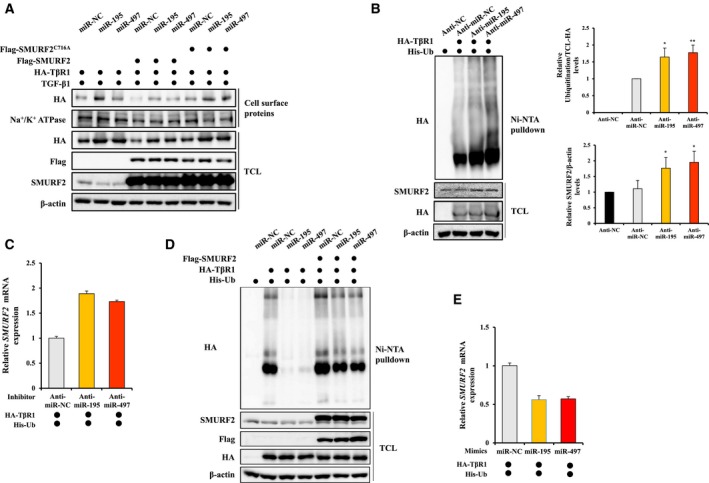
MiR‐195 and miR‐497 regulate cell surface TβRI ubiquitination by modulating SMURF2 levels. (A) A549 cells were co‐transfected with HA‐TβRI, Flag‐SMURF2, Flag‐SMURF2^C716A^, and the mimic of miR‐NC, miR‐195 or miR‐497 for 48 h and then treated with 5 ng·mL^−1^ TGF‐β1 for 4 h. Cell surface proteins were labeled with biotin and purified with avidin‐agarose beads and then analyzed by western blot with anti‐HA antibody. Na^+^/K^+^ ATPase was used as a marker of membrane fraction. (B) HA‐TβRI and His‐Ub were co‐transfected into HEK293T cells, together with the inhibitor for miR‐NC, miR‐195 or miR‐497. After 48 h, HEK293T cells were treated with proteasome inhibitor MG132 (10 µm) for 4 h. HA‐TβRI ubiquitination was detected by immunoprecipitation with the Ni‐NTA agarose pull‐down assay, and total cell lysates were analyzed by immunoblotting (left). Quantification of HA‐TβRI ubiquitination levels was done considering the amount of TCL‐HA TβRI and SMURF2 levels normalized to β‐actin (right). A one‐way ANOVA with Dunnett’s multiple comparison test was used for statistical analysis. The data represent mean ± SEM: *P ≤ 0.05, **P ≤ 0.01 versus control. (C) HEK293T cells were transfected with the inhibitor for miR‐NC, miR‐195 or miR‐497, and *SMURF2* gene expression was then determined by qRT‐PCR. (D) HEK293T cells were co‐transfected with HA‐TβRI, His‐Ub and the mimic for miR‐NC, miR‐195 or miR‐497, in combination with the Flag‐SMURF2 expression vector, and TβRI ubiquitination was then detected. β‐Actin was used as a loading control. (E) HEK293T cells were transfected with the mimic for miR‐NC, miR‐195 or miR‐497, and *SMURF2* gene expression was then determined by qRT‐PCR.

Because SMURF2 is known to be a negative regulator of TGF‐β signaling through ubiquitin‐dependent degradation of activated TβRI, we questioned whether miR‐195 and miR‐497 could regulate the function of SMURF2 as an E3 ligase (Huang and Chen, 2012). Thus, we investigated whether the regulation of SMURF2 by miR‐195 and miR‐497 affects the TβRI levels in A549 cells. Both miR‐195 and miR‐497 mimics increased endogenous TβRI levels in the presence of TGF‐β1 (Fig. S3A). To identify whether the induction of TβRI levels by two miRNA is dependent on miR‐195‐ and miR‐497‐reduced SMURF2 levels, we examined cell surface TβRI levels after transfection with HA‐TβRI, mimics of miR‐195 or miR‐497, and Flag‐SMURF2 WT or SMURF2 catalytic inactive mutant SMURF2^C716A^. After treatment with TGF‐β1, cell surface proteins were isolated and then immunoblotted. Figure 3A shows that the miR‐195 and miR‐497 mimics upregulated TβRI protein levels in both the cell surface and the total cell lysates. Two miRNA‐mediated induction of the TβRI levels was decreased by the overexpressed SMURF2‐WT but not by the overexpressed SMURF2^C716A^. This result suggests that miR‐195 and miR‐497 reduce the levels of endogenous SMURF2, therefore regulating the TβRI levels. On the other hand, the inhibitors of miR‐195 or miR‐497 reduced the cell surface TβRI levels (Fig. S3B).

Polyubiquitination of TβRI has been reported to induce its turnover and degradation (Di Guglielmo *et al.*, 2003). To examine whether miR‐195 and miR‐497 can regulate the stability of TβRI by modulating the SMURF2‐induced ubiquitination, the TβRI ubiquitination pattern was visualized by immunoblotting. HEK293T cells treated with inhibitors of miR‐NC, miR‐195 or miR‐497 were co‐transfected with the HA‐TβRI and His‐Ub vectors followed by the determination of TβRI ubiquitination using the Ni‐NTA Pulldown Assay. The transfection efficiency was confirmed using qRT‐PCR (Fig. S4A,B). Figure 3B shows that TβRI ubiquitination was significantly induced in the miR‐195 and miR‐497 inhibitor‐treated HEK293T cells. In addition, we observed that the *SMURF2* mRNA level was increased (Fig. 3C), whereas the overexpression of miR‐195 and miR‐497 decreased the TβRI ubiquitination and *SMURF2* mRNA level (Fig. 3D,E). Endogenous TβRI ubiquitination was also decreased by two miRNA mimics (Fig. S3C). To test whether the changes in the TβRI ubiquitination by miR‐195 and miR‐497 are dependent on SMURF2, we assessed the TβRI ubiquitination in the presence or absence of SMURF2, which induces TβRI degradation. Flag‐tagged SMURF2 vectors were transfected into HEK293T cells treated with miR‐195 and miR‐497 mimics, and Ni‐NTA was used to pull‐down the modified proteins. As expected, the overexpression of SMURF2 resulted in the recovery of TβRI ubiquitination reduced by the miR‐195 and miR‐497 mimics (Fig. 3D). These results suggest that the miR‐195 and miR‐497 negatively modulate the SMURF2 level and SMURF2‐induced ubiquitination, thereby controlling the cell surface TβRI degradation.

### 3.4 The activation of TGF‐β signaling is regulated by miR‐195 and miR‐497

**Figure 4:**
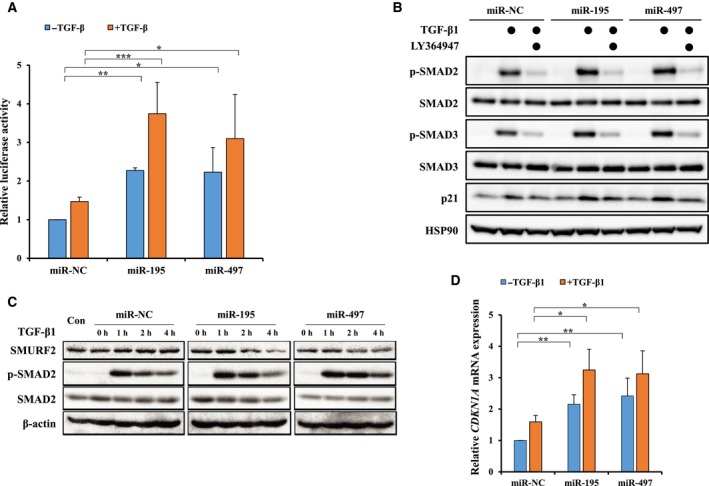
MiR‐195 and miR‐497 regulate the activation of TGF‐β signaling. (A) A549 cells were co‐transfected with the mimic for miR‐NC, miR‐195 or miR‐497, together with the SBE‐luc vector, a TGF‐β‐responsive SMAD luciferase reporter. After 24 h, the luciferase reporter activity was measured after the cells were treated with or without 5 ng·mL^−1^ TGF‐β1 for 24 h. Data are represented as the mean ratios of Renilla to Firefly luciferase activity and are normalized relative to the negative control. A one‐way ANOVA with Dunnett’s multiple comparison test was used for statistical analysis: *P ≤ 0.05, **P ≤ 0.01, ***P ≤ 0.001 versus control. (B) Western blot analysis was done to measure the change in p‐SMAD2 and p‐SMAD3. A549 cells were transfected with the mimic for miR‐NC, miR‐195 or miR‐497 for 24 h and then treated with or without 100 ng·mL^−1^ LY364947. After 24 h, 5 ng·mL^−1^ TGF‐β1 were treated for 4 h. Cell lysates were immunoblotted with anti‐p‐SMAD2, SMAD2, p‐SMAD3, SMAD3 and p21 antibodies. The β‐actin served as a loading control. (C) The p‐SMAD2 expression was detected by western blot. A549 cells were transfected with the mimic for miR‐NC, miR‐195 or miR‐497 and then treated with 5 ng·mL^−1^ TGF‐β1 for 0–4 h. (D) *CDKN1A* gene expression was determined by qRT‐PCR. A549 cells were transfected with the mimic for miR‐NC, miR‐195 or miR‐497 and then treated with or without 5 ng·mL^−1^ TGF‐β1 for 4 h. A two‐way ANOVA with Bonferroni post‐test was used for statistical analysis. The data represent mean ± SEM: *P ≤ 0.05, **P ≤ 0.01, ***P ≤ 0.001.

Our data have shown that miR‐195 and miR‐497 regulate TβRI degradation by modulating SMURF2‐induced ubiquitination. Next, we examined whether miR‐195 and miR‐497 can also regulate the activation of TGF‐β signaling. The TGF‐β‐responsive Cignal SMAD luciferase reporter (Qiagen) containing the SBE was transiently transfected into A549 cells to determine quantitatively the sensitivity of the TGF‐β signaling. Figure 4A shows that the luciferase activity was higher in the miR‐195 and miR‐497 transfected cells than in the negative control. Upon exposure to 5 ng·mL^−1^ TGF‐β1 for 24 h, the TGF‐β1‐induced luciferase activities in the cells transfected with the miR‐195 or miR‐497 mimics were significantly higher than in the cells transfected with miR‐NC. In addition, the mimic of miR‐195 or miR‐497 induced the phosphorylation of SMAD2 and SMAD3 in A549 cells treated with TGF‐β1. However, this effect disappeared after treatment with TGF‐β signaling kinase inhibitor LY364947 (Fig. 4B). Thus, these data indicate that the increase in phosphorylation of SMAD2 and SMAD3 by two miRNA mimics occurs via the activation of the canonical TGF‐β‐SMAD signaling pathway. In contrast, following transfection with anti‐miR‐195 and anti‐miR‐497, the TGF‐β signaling pathway was slightly inhibited, which was reflected by the increased expression of SMURF2 and decreased phosphorylation of SMAD2 and SMAD3 compared with the negative control (Fig. S5A). We also observed that TGF‐β1 stimulation induced a time‐dependent phosphorylation of SMAD2. As shown in Fig. 4C, the peak levels for TGF‐β1‐induced SMAD2 phosphorylation were reached after 1 h stimulation. Phospho‐SMAD2 levels were decreased after a 2‐h TGF‐β1 treatment of A549 cells transfected with miR‐NC. However, TGF‐β1‐induced SMAD2 phosphorylation remained stable over time in the miR‐195 and miR‐497 mimic‐transfected A549 cells. Moreover, we observed that SMURF2 expressions were lower in the miR‐195 and miR‐497 mimic‐transfected A549 cells than in the miR‐NC‐treated A549 cells after 2‐h TGF‐β1 treatment (Fig. 4C). Thus, the TGF‐β1‐induced SMAD2 phosphorylation was prolonged in the miR‐195 and miR‐497 mimic‐transfected cells.

We next measured the expression of TGF‐β target genes such as cyclin‐dependent kinase inhibitor 1A (*CDKN1A*) in A549 transfected with inhibitors or mimics of miR‐NC, miR‐195 and miR‐497. *CDKN1A* acts as a regulator of cell cycle progression of the G1 and S phase (Waldman *et al.*, 1995). To exclude the possibility of direct targeting of CDKN1A by miR‐195 and miR‐497, we examined this possibility using miRNA target prediction databases such as TargetScan, miRanda and miRWalk 2.0 but the results of the target prediction databases showed no binding sites for miR‐195 and miR‐497 in the 3′‐UTR of *CDKN1A*. Figure S5B shows that anti‐miR‐195 and anti‐miR‐497 significantly repressed the TGF‐β1‐induced *CDKN1A* gene; however, the overexpression of miR‐195 and miR‐497 by mimics induced *CDKN1A* expression (Fig. 4D). Moreover, the p21 protein levels were regulated by the mimics and inhibitors of miR‐195 and miR‐497 (Figs 4C and S5C). Indeed, miR‐195‐ and miR‐497‐mediated induction of p21 protein levels was decreased by LY364947 treatment (Fig. 4B). These results demonstrate that the sustained activation of TGF‐β signaling by miR‐195 and miR‐497 enhanced the activity and expression of TGF‐β target genes. Taken together, these data suggest that miR‐195 and miR‐497 function as positive regulators of TGF‐β signaling by inhibiting SMURF2‐dependent TβRI ubiquitination.

MiR‐195 and miR‐497 are located on the same chromosomal locus, and these miRNA are grouped into the same family based on sequence similarity (Flavin *et al.*, 2009). Hence, we expected that these miRNA might act synergistically to inhibit the *SMURF2* mRNA simultaneously. However, in our experiments, we were not able to observe any synergistic effects of co‐transfection with two miRNA on the inhibition of *SMURF2* gene and *p21* gene (Fig. S6A,B).

### 3.5 MiR‐195 and miR‐497 regulate proliferation, colony formation and invasion

**Figure 5:**
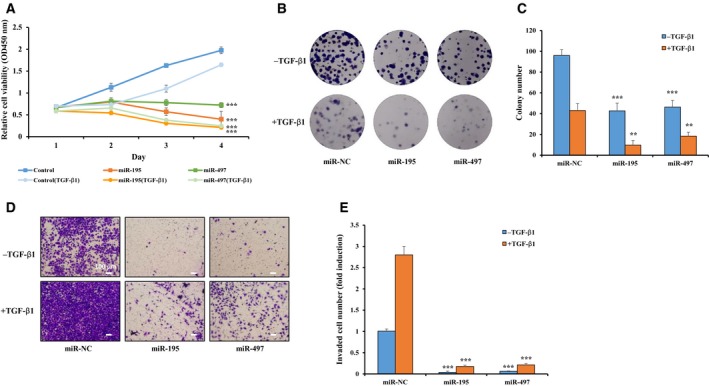
The effects of miR‐195 and miR‐497 on A549 cell viability, colony formation and invasion. (A) A549 cells were transfected with the mimic for miR‐NC, miR‐195 or miR‐497 and then treated with or without 1 ng·mL^−1^ TGF‐β1. Cell viability was measured at the time indicated by the WST assay. (B,C) Colony formation assay of the A549 cells were transfected with the mimic for miR‐NC, miR‐195 or miR‐497 and then treated or not treated with 1 ng·mL^−1^ TGF‐β1. Cells were cultured for 10 days and stained with crystal violet in a six‐well plate. (D,E) Matrigel invasion assay was used to check the invasive ability of the cells transfected with miR‐195 or miR‐497 mimic with or without 1 ng·mL^−1^ TGF‐β1 (10× magnification). Scale bars: 100 µm. The cells that invaded through the Matrigel were fixed and stained with Giemsa. The number of invaded cells for each group was normalized to the control. A one‐way ANOVA with Dunnett’s multiple comparison test was used for statistical analysis: **P ≤ 0.01, ***P ≤ 0.001 versus control.

To determine whether miR‐195 and miR‐497 influence the cancer phenotype of A549 cells, we carried out a series of biological experiments to determine the effects of miR‐195 and miR‐497 on cancer cell phenotypes. First, cell viability was assessed 24, 48 and 72 h after transfection of the inhibitors and mimics. The WST assay revealed that the A549 cells transfected with the mimics of miR‐195 and miR‐497 had a dramatically inhibited cell proliferation compared with the negative control both in the absence and in the presence of TGF‐β1 (Fig. 5A). Conversely, A549 cells transfected with the inhibitor of miR‐195 or miR‐497 showed a significant increase in cell proliferation (Fig. S7A). Next, to verify whether the effect of miR‐195 or miR‐497 on the cell proliferation is caused by the reduction of SMURF2 levels, we co‐transfected A549 cells with the mimic of miR‐195 or miR‐497 alone, or together with Flag‐SMURF2 with TGF‐β1. As expected, reduction of cell proliferation by miR‐195 or miR‐497 was restored by the overexpression of SMURF2, indicating that miR‐195 and miR‐497 inhibit cell proliferation by reducing SMURF2 levels in lung cancer cells (Fig. S8A). Lastly, to identify whether activation of the TGF‐β signaling via decrease in SMURF2 levels contributes to the ability of these miRNA to suppress cell viability, we repeated this WST assay with the TGF‐β signaling inhibitor, LY364947. In control cells, TGF‐β1‐induced suppression of cell proliferation was restored by treatment with LY364947 and this response also occurred identically with the mimic of miR‐195‐ or miR‐497‐transfected cells (Fig. S9A).

Several studies have reported on miR‐195 and miR‐497 as tumor suppressors. Therefore, we measured the effects of miR‐195 and miR‐497 on the cell growth characteristics by colony formation assay. The results show that the miR‐195 and miR‐497 mimics reduced the colony formation ability of the A549 cells compared with the negative control; these responses also occurred identically in the presence of TGF‐β1 (Fig. 5B,C). In contrast, anti‐miR‐195 or anti‐miR‐497 enhanced the colony formation ability (Fig. S7B,C). These data show that miR‐195 and miR‐497 inhibit cell proliferation and cell survival through activating TGF‐β signaling by suppression of SMURF2‐induced TβRI ubiquitination.

The Matrigel invasion assay showed that miR‐195 and miR‐497 mimics reduced the invasive ability of lung cancer cells compared with control. Interestingly, as known TGF‐β1 treatment rapidly increased invasive cells, but miR‐195 and miR‐497 mimics still reduced the amount of invading cells compared with control in the presence of TGF‐β1 (Fig. 5D,E). Indeed, when transfecting with anti‐miR‐195 or anti‐miR‐497, the amount of invaded cells was increased by both two anti‐miRNA compared with the control (Fig. S7D,E). In addition, despite the inhibition of TGF‐β signaling by LY364947 treatment, the invasive effects of the cells transfected with two miRNA were also decreased compared with control cells, suggesting that TGF‐β signaling does not contribute significantly to the effects of these miRNA on cell invasion (Fig. S9B,C). In fact, the reduction of SMURF2 levels by siRNA or inhibitors leads to a decrease not only in cell survival/proliferation but also in migration and invasive abilities of cancer cells (David *et al.*, 2014; Jin *et al.*, 2009; Klupp *et al.*, 2019). Hence, it is possible that miR‐195 or miR‐497 could decrease invasiveness of lung cancer cells by suppressing the expression of *SMURF2* gene directly, although TGF‐β signaling was activated. We demonstrated this possibility by performing invasion assay with Flag‐SMURF2; ectopically expressed SMURF2 increased the invasion ability of lung cancer cells reduced by the two miRNA in the presence of TGF‐β1 (Fig. S8B,C). We also checked SMURF2 protein levels by the overexpression the mimic of miR‐195 or miR‐497, or Flag‐SMURF2 (Fig. S8D). Thus, miR‐195 and miR‐497 might regulate cell invasion in a SMURF2‐dependent manner rather than the activation of TGF‐β signaling. Taken together, these results suggest that miR‐195 and miR‐497 have an important role as a potential tumor‐suppressor in lung cancer by inhibiting cell growth and invasion.

### 3.6 Overexpression of miR‐195 and miR‐497 suppresses xenograft tumor growth

**Figure 6:**
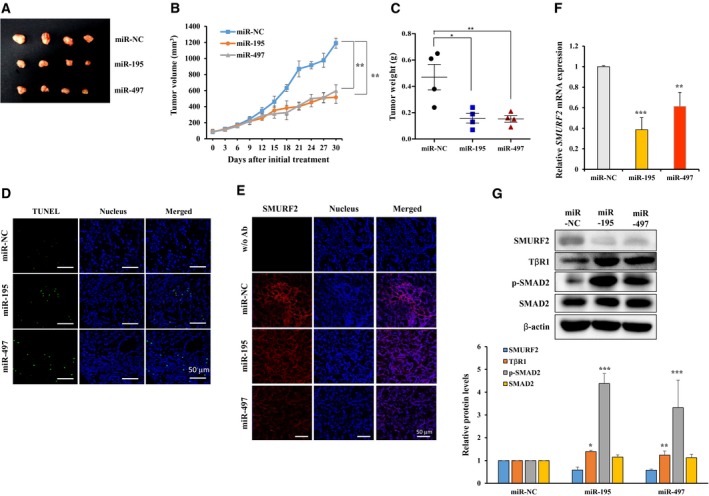
MiR‐195 and miR‐497 suppress tumor growth in an A549 xenograft model. (A) Effects of miR‐195 and miR‐497 on tumor formation in a nude mouse xenograft model. Representative tumor sizes for each group of nude mice. (B) Growth curve of the tumor volumes was measured at the indicated times after tumor cell inoculation. (C) Tumor weight of the xenograft tumors. (D) TUNEL staining detected cell apoptosis in the xenograft tumors. Blue staining represents the nucleus, and green staining TUNEL‐positive cells. Scale bars: 50 µm. (E) immunofluorescence staining of SMURF2 was measured in tumor tissues treated with miR‐195 or miR‐497. (F,G) the expressions of *SMURF2* mRNA and protein were determined in xenograft tumors treated with miR‐195 or miR‐497. Quantification of SMURF2, TβRI, p‐SMAD2, and SMAD2 levels was done considering the amount of β‐actin protein in each case (G, bottom). A one‐way ANOVA with Dunnett’s multiple comparison test was used for statistical analysis: *P ≤ 0.05, **P ≤ 0.01, ***P ≤ 0.001 versus control.

To determine the role of miR‐195 and miR‐497 in a xenograft nude mouse model, we subcutaneously inoculated nude mice with A549 cells with miR‐NC, miR‐195 and miR‐497 mimics. The mice were sacrificed 30 days after the inoculation, and the tumors were resected and weighed. The results show that the overexpression group of miR‐195 and miR‐497 significantly suppressed the tumor size by 50% compared with miR‐NC (Fig. 6A) and decreased the tumor volume and weight (Fig. 6B,C). Furthermore, TUNEL assay showed that miR‐195 and miR‐497 significantly increased the apoptosis rate of the xenograft tumors of mice transfected with miR‐195 or miR‐497 compared with the control (Fig. 6D). To assess whether miR‐195 and miR‐497 could regulate SMURF2 expression *in vivo*, we examined the expression levels of SMURF2. Both protein levels and mRNA levels of SMURF2 were reduced in miR‐195 and miR‐497 overexpressed tumor tissues compared with the control (Fig. 6E–G). In contrast to SMURF2 levels, TβRI and p‐SMAD2 expression was significantly upregulated in the xenograft tumors of mice transfected with the miR‐195 or miR‐497 mimic compared with the control (Fig. 6G). These results indicate that overexpression of miR‐195 and miR‐497 significantly suppresses tumor growth *in vivo*.

## 4 Discussion

In this study, we analyzed the expressions of miR‐195 and miR‐497 in the tissue and blood of lung cancer patients in the NCBI GEO database (http://www.ncbi.nlm.nih.gov/geo/query/acc.cgi?acc=GSE51853 and http://www.ncbi.nlm.nih.gov/geo/query/acc.cgi?acc=GSE1768) and in several lung cancer cell lines. We found that miR‐195 and miR‐497 were significantly downregulated in lung cancer. Our results demonstrate that ectopic expression of miR‐195 and miR‐497 significantly suppressed lung cancer cell proliferation, colony formation and invasion in the cancer cells and tumor growth in a xenograft mouse model. These findings indicate that miR‐195 and miR‐497 might act as tumor suppressors whose downregulation may contribute to the progression and prognosis of cancer. Some studies have shown that miR‐195 and miR‐497 have different functions in other types of cancer. MiR‐195 expression in RCC was upregulated, and it promoted cell proliferation, migration and invasion in human RCC cell lines (Jin *et al.*, 2017). In addition, miR‐497 was upregulated in pancreatic cancer (Bloomston *et al.*, 2007). However, most studies have reported that miR‐195 and miR‐497 function as tumor suppressors in many types of cancer, consistent with our findings (Furuta *et al.*, 2013; Li *et al.*, 2011). To identify the molecular mechanism of miR‐195 and miR‐497 in cancer, we selected the *SMURF2* gene among the components of TGF‐β signaling as putative targets of miR‐195 and miR‐497 using prediction databases. In fact, many studies have reported that TGF‐β signaling promotes miR‐195 and miR‐497 expression (Chen *et al.*, 2016; Duan and Chen, 2016; Song *et al.*, 2017), and miR‐195/497 targets other proteins associated with TGF‐β signaling. We analyzed the expression of the *SMURF2* gene in tumor and paired normal lung tissues from primary NSCLC patients in the NCBI GEO database (http://www.ncbi.nlm.nih.gov/geo/query/acc.cgi?acc=GSE21933) and lung cancer cell lines. We found that *SMURF2* expression was increased in lung cancer.

SMURF2 is a ubiquitin E3 ligase and has a crucial role in the degradation of numerous proteins by 26S proteasomes (ten Dijke and Hill, 2004). SMURF2 has gained prominence because it induces the ubiquitination and degradation of the components of TGF‐β signaling (Fukasawa *et al.*, 2004; Nakano *et al.*, 2009). Moreover, the SMURF2 and SMAD7 complex binds to the activated receptor complex and subsequently regulates the endocytosis, trafficking and downregulation of cell surface TβRI. The regulation of TβRI by SMURF2 affects the capacity of cells to respond to the TGF‐β signal. Recent studies have also reported that the *SMURF2* gene was inhibited by miR‐322/503 in the intestinal epithelial cell (Cao *et al.*, 2014) and by miR‐15b in pancreatic cancer (Zhang *et al.*, 2015). However, it is not clear yet which miRNA regulate the abundance of SMURF2 in lung cancer. Therefore, we focused on the effects of miR‐195 and miR‐497 on the *SMURF2* gene expression and their biological functions in lung cancer. In our experiments, we observed that miR‐195 and miR‐497 downregulate the *SMURF2* gene expression by direct targeting of the 3′‐UTR of *SMURF2* using the luciferase reporter assay.

Many studies have reported that perturbation of TGF‐β signaling is often considered a pathogenic factor in tumor progression due to its tumor‐promoting effects or tumor suppressor effects (Butz *et al.*, 2012; Ikushima and Miyazono, 2010). TGF‐β signaling has two distinct and opposite roles in metastasis and cancer progression depending on the stage of cancer (Zarzynska, 2014). These dramatic changes in TGF‐β signaling reflect a variety of dynamic alterations that occur within cancer cells. Under normal conditions, TGF‐β signaling is tightly regulated by numerous components. However, perturbations of this balance induce the transformation from normal cells to cancer cells. The ubiquitin‐proteasome pathways are also important in tightly regulating TGF‐β signaling by modulating TβRI. This pathway requires E1, E2 and E3 ligases together with the SMAD7 adaptor protein. Phosphorylated TβRI interacts with several types of E3 ligases, SMURF1, SMURF2, WWP1 or NEDD4‐2, which were reported as regulators of TβRI stability. The formation of the TβRI‐SMAD7 complex enhances the ubiquitination‐dependent degradation of TβRI. In contrast, USP4 or USP15 removes SMURF2‐mediated ubiquitination of TβRI and thus stabilizes the TβRI (Eichhorn *et al.*, 2012; Zhang *et al.*, 2012).

The abundance of cell surface TβRI is regulated by clathrin‐dependent or lipid‐raft‐caveolar endocytic pathways. Internalization of TGF‐β receptor by clathrin‐dependent endocytosis to EEA1‐positive endosomes promotes TGF‐β signaling. Endofin and SARA are enriched in EEA1‐positive endosomes and facilitate the formation of the SMAD complex and the activation of R‐SMADs. Moreover, the internalized receptors can be recycled and returned to the membrane in a Rab11‐dependent manner. Meanwhile, TβRI internalization by the lipid‐raft‐caveolar pathway effectively decreasing or abolishing TGF‐β signaling, causes the recruitment of SMURF2 and SMAD7. The SMURF2 and SMAD7 complex initiates the proteasome degradation of TβRI and rapid turnover (Del Galdo *et al.*, 2008; Huang and Chen, 2012). Our data suggest that miR‐195 and miR‐497 may protect TβRI from the lipid raft‐caveolar pathway and the SMURF2‐mediated degradation, whereas downregulation of miR‐195 and miR‐497 may promote this ubiquitination and degradation.

**Figure 7:**
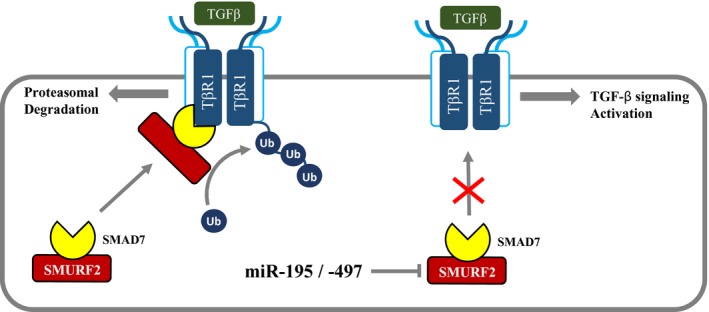
Working model of miR‐195‐ and miR‐497‐mediated regulation of TGF‐β signaling by targeting SMURF2 in early‐stage lung cancer. MiR‐195 and miR‐497 negatively regulate *SMURF2* expression and SMURF2‐dependent TβRI ubiquitination, thereby increasing the activation of TGF‐β signaling.

Although miR‐195 and miR‐497 did not directly regulate the TβRI expression, they can upregulate cell surface TβRI expression by regulation of SMURF2. We demonstrated that ectopic expression of miR‐195 and miR‐497 by mimics suppressed TβRI ubiquitination and degradation through the repression of *SMURF2* gene expression. Moreover, upregulation of SMURF2 by the SMURF2‐WT expression vector recovered TβRI ubiquitination. These results suggest that the reduction of the *SMURF2* gene expression by miR‐195 or miR‐497, resulting in the activation of TGF‐β signaling, has an important role in inhibiting early‐stage lung cancer (Fig. 7). However, two miRNA still reduced the invasion ability of lung cancer cells, whereas TGF‐β1 rather increased the amount of invasive cells. This difference might have been the result of the different roles of TGF‐β1 and SMURF2 in late‐stage cancer. TGF‐β1 increases migration and invasion of the cells during metastasis, but the reduction of SMURF2 by siRNA or inhibitors leads to a decrease in the invasion ability of cancer cell. Subsequent studies are required to identify accurately the contribution of TGF‐β signaling to the functions of miR‐195 and miR‐497 during metastasis. These results expand the understanding of the association between post‐transcriptional regulation by miRNA and the SMURF2‐dependent ubiquitin system.

In fact, miR‐195 expression is known to affect the TGF‐β signaling pathway by targeting *SMAD2* and *SMAD7* genes in glioma–U87 cell line (Duan and Chen, 2016). Also, miR‐497 is known to target SMAD7 in breast cancer (Liu *et al*, 2016). Based on the previous and the current studies, miR‐195 and miR‐497 would reduce TβRI ubiquitination in two ways: downregulation of SMURF2 and downregulation of SMAD7 by miRNA. We intend to test further the reduction of TβRI ubiquitination by SMAD7 using two miRNA.

## 5 Conclusion

In conclusion, this study identified a group of miRNA predicted to be downregulated in lung cancer patients and selected the miR‐195 and miR‐497 cluster. We demonstrated that miR‐195 and miR‐497 directly target *SMURF2* with the luciferase assay and regulate TGF‐β signaling in lung cancer cells. This regulation prolonged TGF‐β‐induced R‐SMAD activation by inhibiting the *SMURF2* gene. As a result, we observed that activated TGF‐β signaling by miR‐195 and miR‐497 significantly suppressed tumor growth *in vitro* and *in vivo*. However, miR‐195 and miR‐497 also suppressed invasive ability of lung cancer cells, although the activation of TGF‐β signaling increased cell invasion. This study expands the understanding of the mechanisms for the post‐transcriptional regulation of *SMURF2* by regulation of miR‐195 and miR‐497, and its ability to modulate TGF‐β signaling. Our study sheds light on further clinical benefits of miR‐195 and miR‐497 as diagnosis biomarkers and therapeutic targets for lung cancer.

## Conflict of interest

The authors declare no conflict of interest.

## Author contributions

D‐KC, JP, EB and EJS designed the research. D‐KC, JP, MC and EB performed the research. MJ performed the animal experiment and analyzed the data. YSY, EEK and J‐HB contributed new reagents or analytic tools. D‐KC, JP and EJS wrote the paper.

## Supporting information


**Fig. S1.** The expression of miR‐195, miR‐497 and *SMURF2* gene in lung cancer cell lines.
**Fig. S2.** Evaluation of transfection efficiency in A549 cells.
**Fig. S3.** MiR‐195 and miR‐497 regulate the levels and the ubiquitination of TβRI.
**Fig. S4.** Evaluation of transfection efficiency in HEK293T cells.
**Fig. S5.** Anti‐miR‐195 and anti‐miR‐497 inhibit TGF‐β signaling.
**Fig. S6.** MiR‐195 and miR‐497 target *SMURF2* gene and regulate the expression of *p21* gene.
**Fig. S7.** The effects of anti‐miR‐195 and anti‐miR‐497 on cell viability, colony formation and invasion.
**Fig. S8.** The effects of *SMURF2* on miR‐195/497‐mediated reduction of cell proliferation.
**Fig. S9.** The effects of miR‐195 and miR‐497 on cell viability and invasion in treatment with TGF‐β1 and LY364947.
