# Supplementary material for: MiR‐195 and miR‐497 suppress tumorigenesis in lung cancer by inhibiting SMURF2‐induced TGF‐β receptor I ubiquitination
Source: Mol Oncol. 2019 Nov 8;13(12):2663–78. doi: 10.1002/1878-0261.12581 (PMC6887584; doi:10.1002/1878-0261.12581)
Supplement: Supplementary file 1 — Fig. S1. The expression of miR‐195, miR‐497 and SMURF2 gene in lung cancer cell lines. Fig. S2. Evaluation of transfection efficiency in A549 cells. Fig. S3. MiR‐195 and miR‐497 regulate the levels and the ubiquitination of TβRI. Fig. S4. Evaluation of transfection efficiency in HEK293T cells. Fig. S5. Anti‐miR‐195 and anti‐miR‐497 inhibit TGF‐β signaling. Fig. S6. MiR‐195 and miR‐497 target SMURF2 gene and regulate the expression of p21 gene. Fig. S7. The effects of anti‐miR‐195 and anti‐miR‐497 on cell viability, colony formation and invasion. Fig. S8. The effects of SMURF2 on miR‐195/497‐mediated reduction of cell proliferation. Fig. S9. The effects of miR‐195 and miR‐497 on cell viability and invasion in treatment with TGF‐β1 and LY364947. [file MOL2-13-2663-s001.pdf]

Supplementary data

A

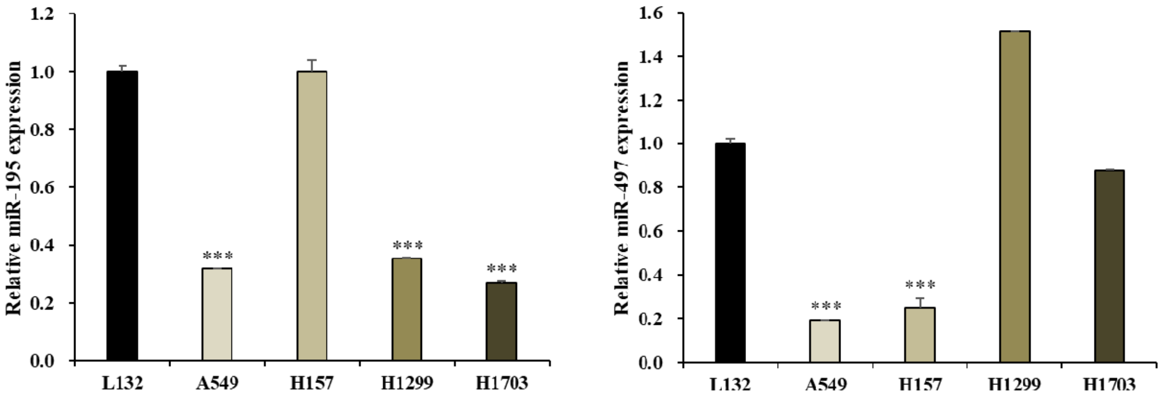

B

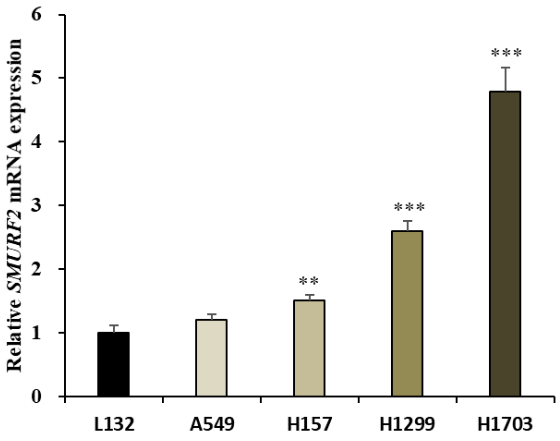

Fig. S1. The expression of miR-195, miR-497, and *SMURF2* gene in lung cancer cell lines

(A, B) Expression levels of miR-195, miR-497, or *SMURF2* gene were measured by qRT-PCR in A549, H157, H1299, H1703 or L132 cell lines. The data are shown as the mean ± SD of three independent experiments (*P < 0.05, **P < 0.005, ***P < 0.001, Two-tailed *t*-test).

A

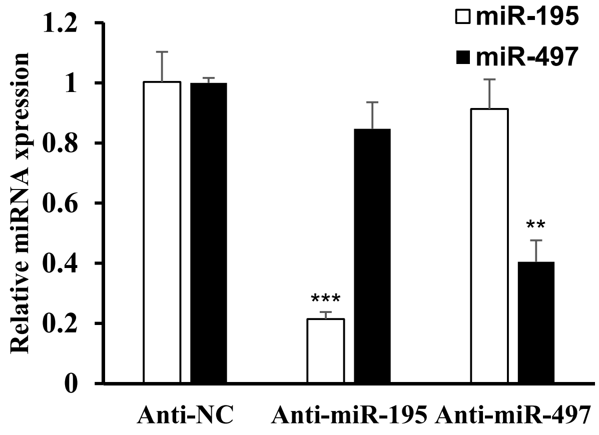

B

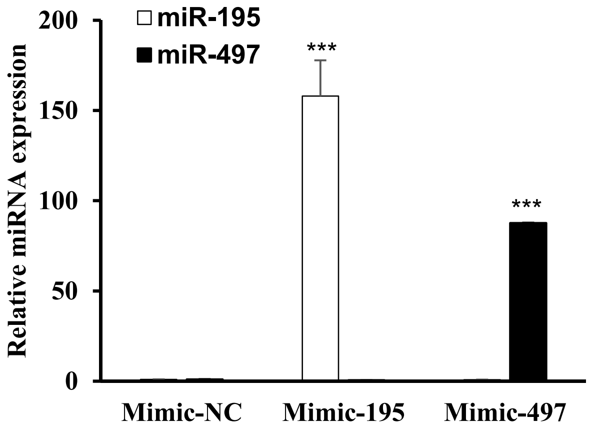

Fig. S2. Evaluation of transfection efficiency in A549 cells. (A, B) The abundance of miR-195 and miR-497 was assessed by qRT-PCR, which detected expressions in A549 cells transfected with the inhibitors or mimics for miR-NC, miR-195 and miR-497 for 24 h. Expression of miR-195 and miR-497 was normalized to U6 snRNA. A one-way ANOVA with Dunnett's multiple comparison test was used for statistical analysis, $*P \leq 0.05$, $**P \leq 0.01$, $***P \leq 0.001$ versus control.

A

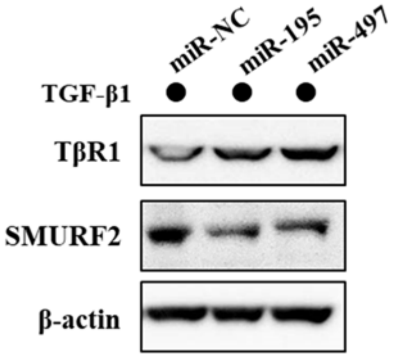

miR-NC
miR-195
miR-497
TGF-β1
TβR1
SMURF2
β-actin

B

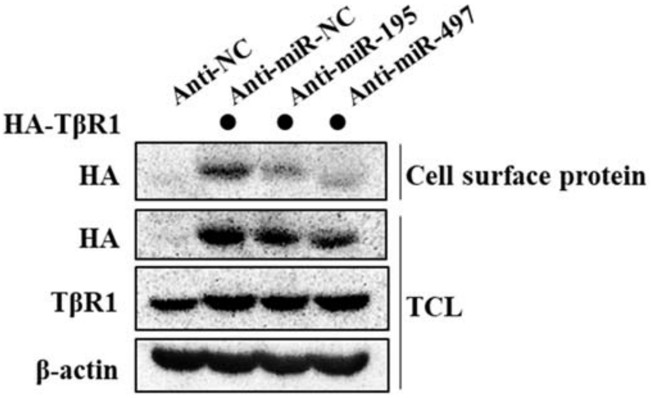

Anti-NC
Anti-miR-NC
Anti-miR-195
Anti-miR-497
HA-TβR1
HA
Cell surface protein
HA
TβR1
TCL
β-actin

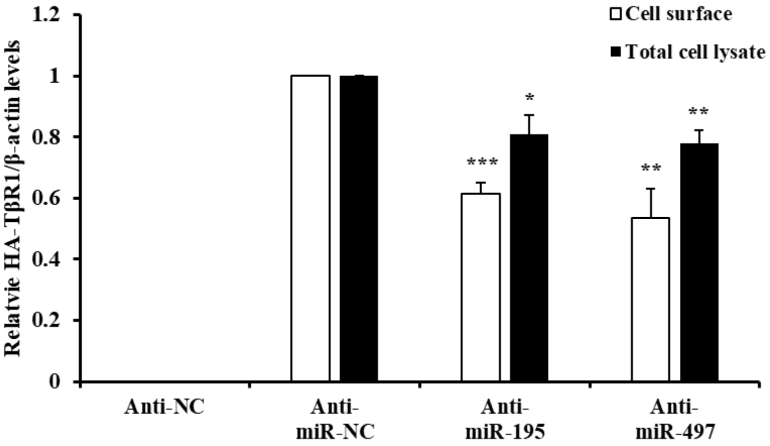

Relatvie HA-TβR1/β-actin levels
1.2
1
0.8
0.6
0.4
0.2
0
Cell surface
Total cell lysate
***
*
**
**
Anti-NC
Anti-
miR-NC
Anti-
miR-195
Anti-
miR-497

C

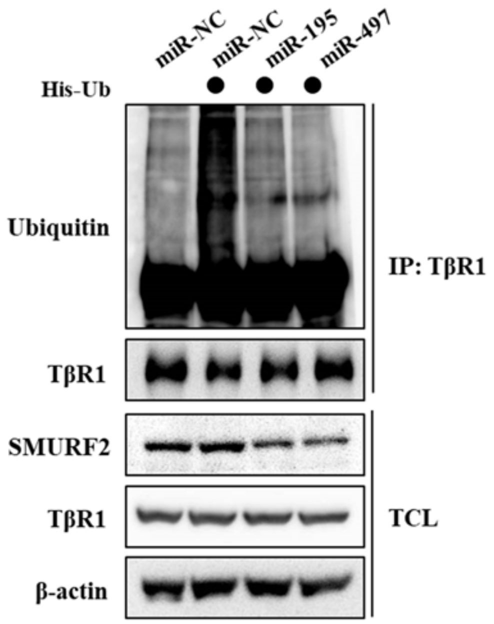

Fig. S3. MiR-195 and miR-497 regulate the levels and the ubiquitination of TβRI. (A) Western blot analysis was done to measure the change in the TβRI levels. A549 cells were transfected with mimics for miR-NC, miR-195 or miR-497 for 48 h and then treated with 5 ng/mL TGF-β1 for 4 h. Cell lysates were immunoblotted with anti-TβRI, SMURF2 antibodies. The β-actin served as a loading control. (B) Western blot analysis of immunoprecipitates and total cell lysates (TCL) derived from A549 cells. Cell surface proteins were labeled with biotin and purified with avidin-agarose beads and then analyzed by Western blot (top). Quantification of cell surface and total cell lysate HA-TβRI expression levels was done considering the amount of β-actin protein in each case (bottom). The data are shown as the mean ± SD of three independent experiments (*P < 0.05, **P < 0.005, ***P < 0.001, Two-tailed *t*-test). (C) HEK293T cells were transfected with His-Ub and the mimics for miR-NC, miR-195, or miR-497. After 48 h, cells were treated with 5 ng/mL TGF-β1 and 10 μM MG132 for 4 h. Endogenous TβRI ubiquitination was detected by immunoprecipitation with an anti-TβRI antibody, and then TCL were analyzed by immunoblotting.

A

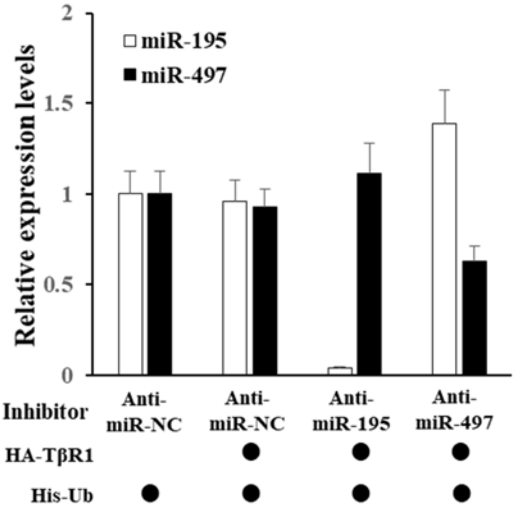

B

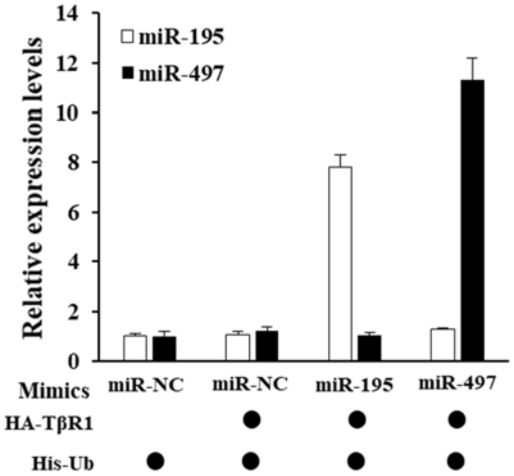

Fig. S4. Evaluation of transfection efficiency in HEK293T cells. (A) MiR-195 and miR-497 expressions from the experiments summarized in Fig. 3B were markedly decreased by transfection with miR-195 or miR-497 inhibitor. (B) The expression level of miR-195 and miR-497 from the experiments summarized in Fig. 3D was determined by qRT-PCR.

A

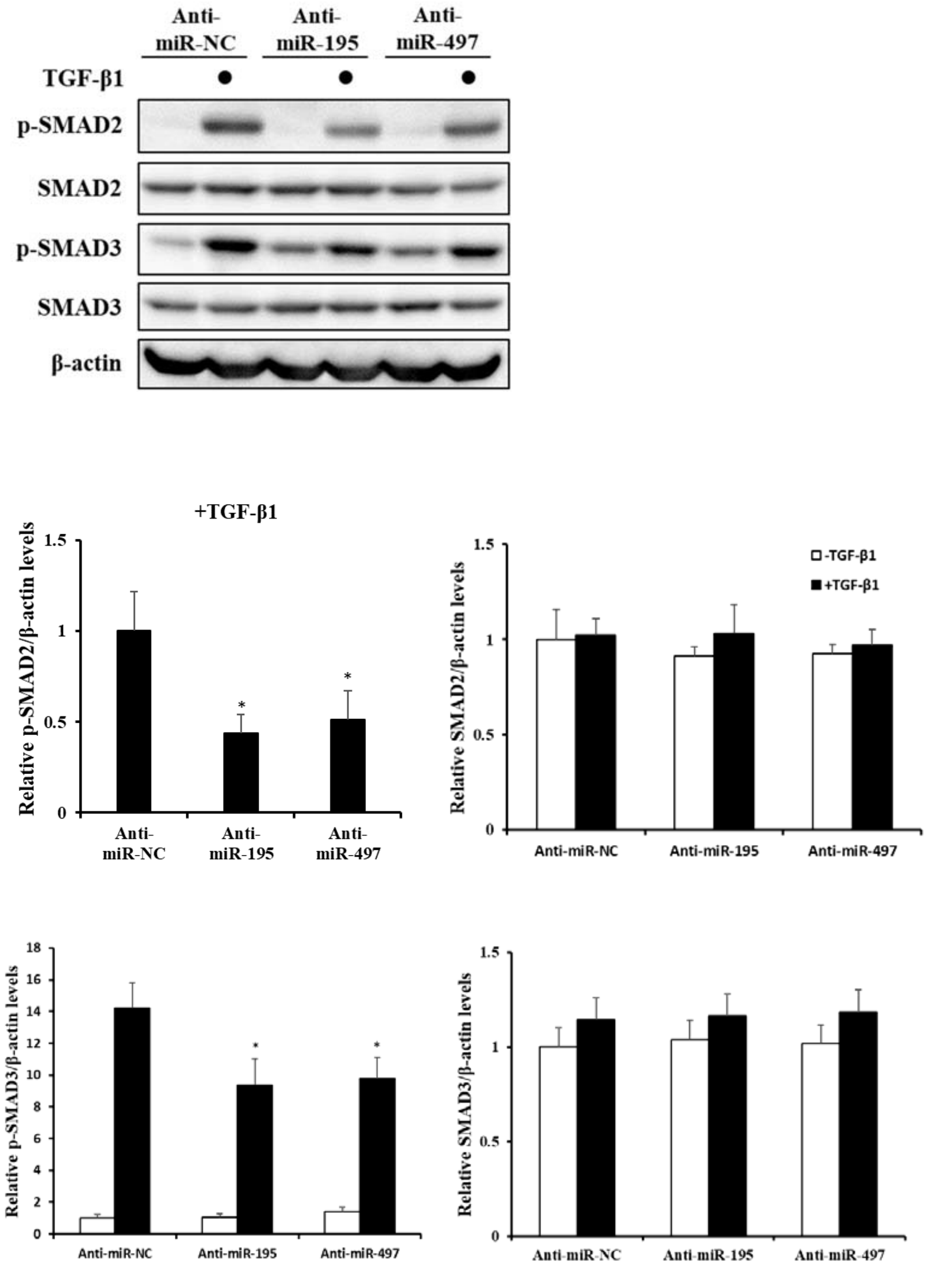

Anti-miR-NC
Anti-miR-195
Anti-miR-497
TGF-β1
p-SMAD2
SMAD2
p-SMAD3
SMAD3
β-actin
+TGF-β1
Relative p-SMAD2/β-actin levels
Relative SMAD2/β-actin levels
-TGF-β1
+TGF-β1
Relative p-SMAD3/β-actin levels
Relative SMAD3/β-actin levels

B

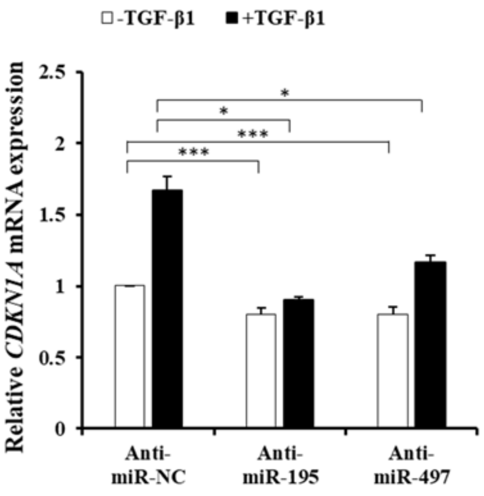

C

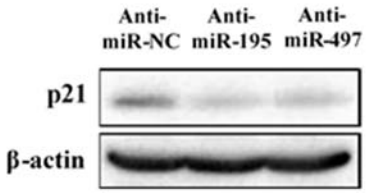

Fig. S5. Anti-miR-195 and miR-497 inhibit TGF-β signaling. (A) Western blot analysis was done to measure the change in the p-SMAD2 and p-SMAD3. A549 cells were transfected with inhibitors for miR-NC, miR-195 or miR-497 for 48 h and then treated with or without 5 ng/mL TGF-β1 for 4 h. Cell lysates were immunoblotted with anti-p-SMAD2, SMAD2, p-SMAD3 and SMAD3 antibodies. The β-actin served as a loading control (top). Quantification of p-SMAD2, SMAD2, p-SMAD3 and SMAD3 expression levels was done considering the amount of β-actin protein in each case (bottom). The data are shown as the mean ± SD of three independent experiments (*$P < 0.05$, Student's t-test). (B) *CDKN1A* gene expression was determined by qRT-PCR. A549 cells were transfected with the inhibitors for miR-NC, miR-195 or miR-497 and then treated with or without 5 ng/mL TGF-β1 for 4 h. A two-way ANOVA with Bonferroni posttest was used for statistical analysis. The data represent mean ± SEM; *$P \leq 0.05$, **$P \leq 0.01$, ***$P \leq 0.001$. (C) Western blot analysis was done to analyze the expression of p21 protein in A549 cells. β-actin served as a loading control.

A

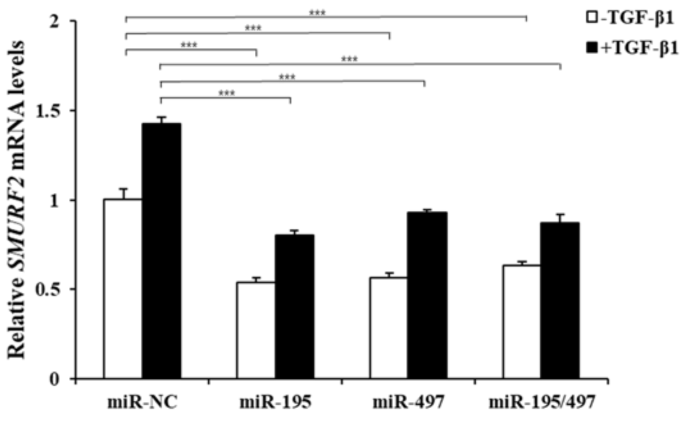

B

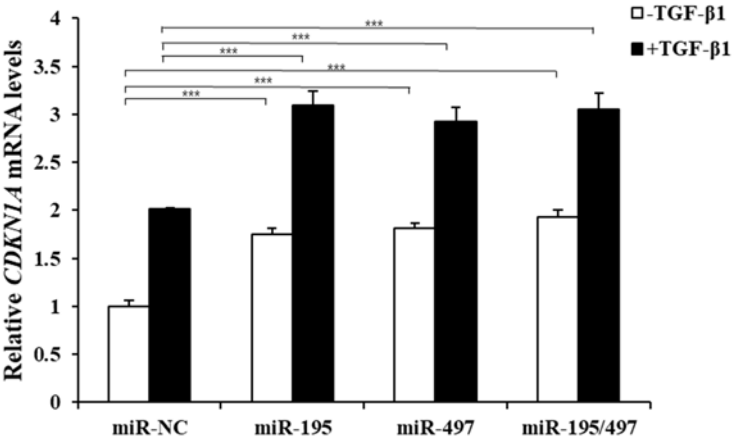

Fig. S6. MiR-195 and miR-497 target *SMURF2* gene and regulate the expression of *p21* gene. (A, B) The expression of *SMURF2* gene or *p21*gene was determined by qRT-PCR. A549 cells were transfected with the mimics for miR-NC, miR-195 or miR-497 alone, or together with miR-195 and miR-497 and then treated with or without 5 ng/mL TGF-β1 for 4 h. A one-way ANOVA with Dunnett's multiple comparison test was used for statistical analysis, *P ≤ 0.05, **P ≤ 0.01, ***P ≤ 0.001 versus control.

A

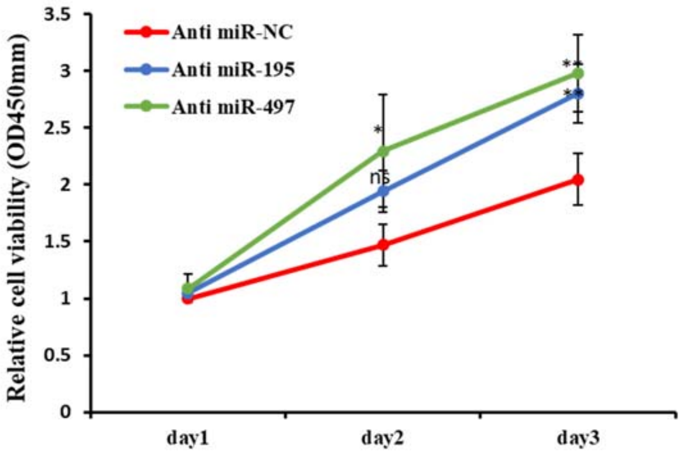

Anti miR-NC
Anti miR-195
Anti miR-497
Relative cell viability (OD450mm)
3.5
3
2.5
2
1.5
1
0.5
0
day1
day2
day3
*
ns
**
**

B

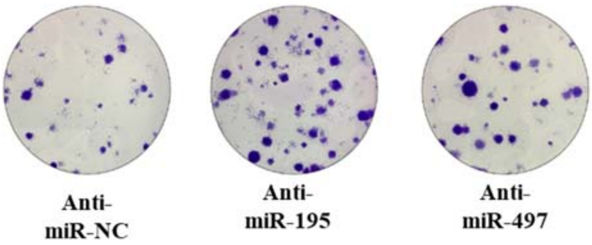

Anti-
miR-NC
Anti-
miR-195
Anti-
miR-497

C

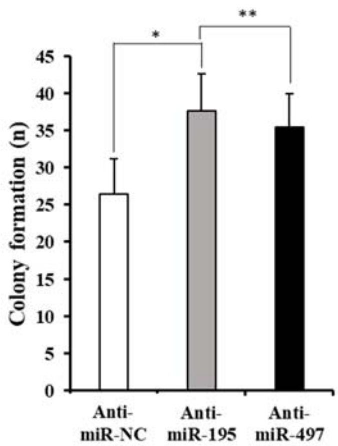

Colony formation (n)
45
40
35
30
25
20
15
10
5
0
*
**
Anti-
miR-NC
Anti-
miR-195
Anti-
miR-497

D

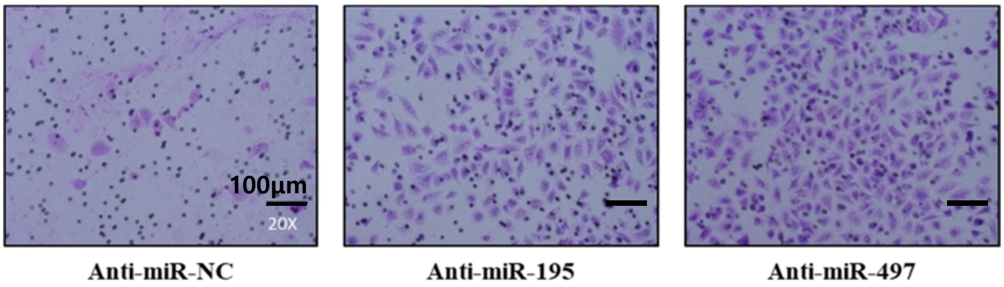

E

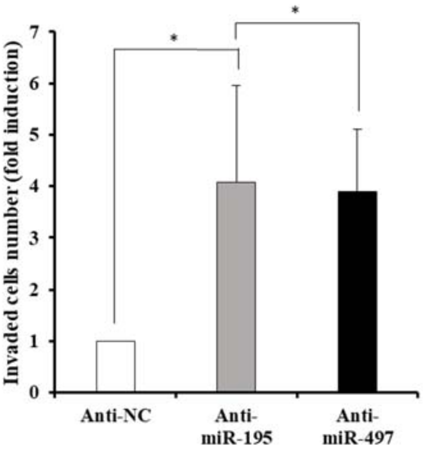

Fig. S7. The effects of anti-miR-195 or miR-497 on cell viability, colony formation and invasion. (A) A549 cells were transfected with the inhibitors for miR-NC, miR-195 or miR-497, and the cell viability was measured by the WST assay. (B, C) Colony formation assay of the A549 cells transfected with the inhibitors for miR-NC, miR-195 or miR-497 for 48 h. Cells were cultured for 10 days and stained with crystal violet in a 6 well plate. (D, E) Matrigel invasion assay was performed to identify the effect of inhibition of miR-195 or miR-497 (20× magnification). Scale bars indicate 100 µm. The cells that invaded through the Matrigel were fixed and stained with giemsa. The number of invaded cells for each group was normalized to the control. A one-way ANOVA with Dunnett's multiple comparison test was used for statistical analysis, $*P \leq 0.05$, $**P \leq 0.01$ versus control.

A

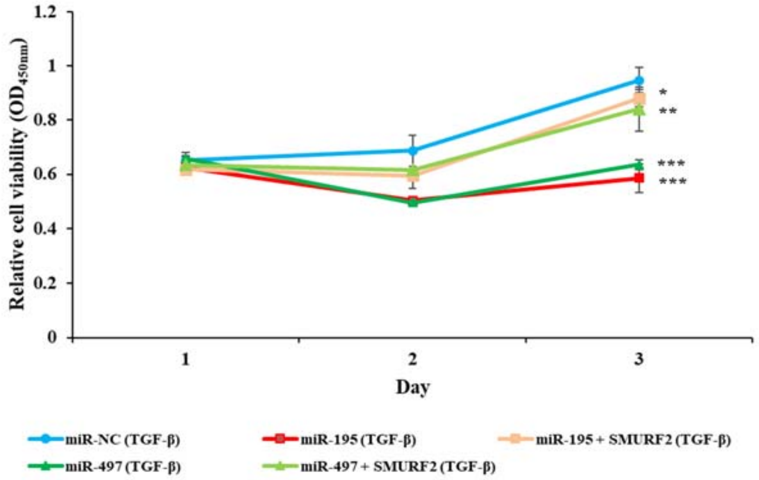
1.2
1
0.8
0.6
0.4
0.2
0
Relative cell viability (OD450nm)
1
2
3
Day
*
**
***
***
miR-NC (TGF-β)
miR-195 (TGF-β)
miR-195 + SMURF2 (TGF-β)
miR-497 (TGF-β)
miR-497 + SMURF2 (TGF-β)

B

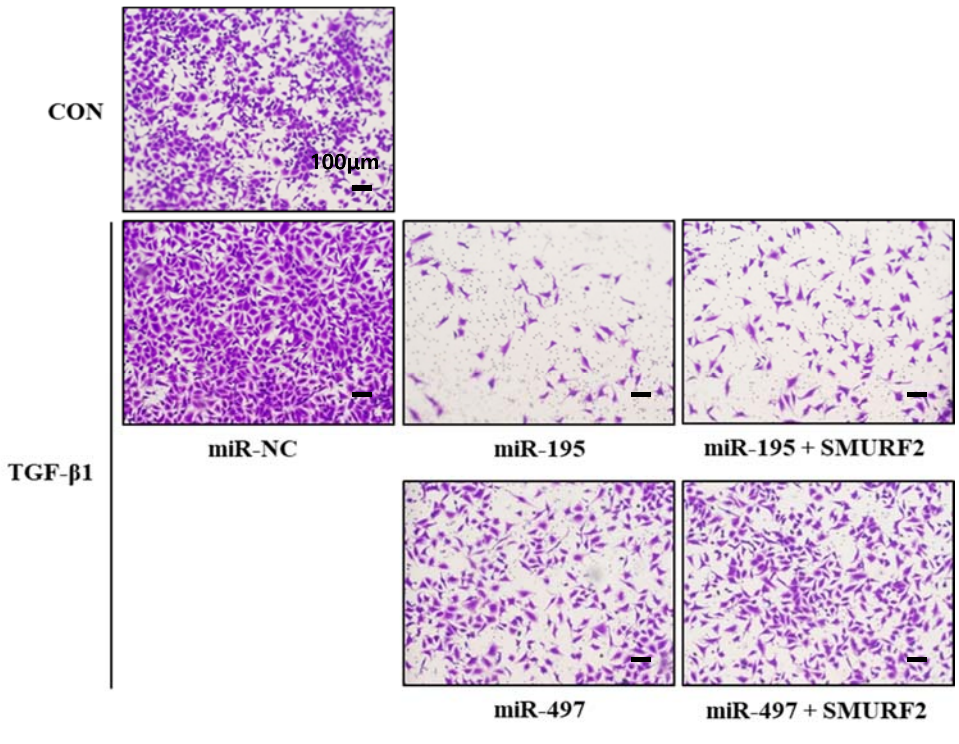
CON
100μm
TGF-β1
miR-NC
miR-195
miR-195 + SMURF2
miR-497
miR-497 + SMURF2

C

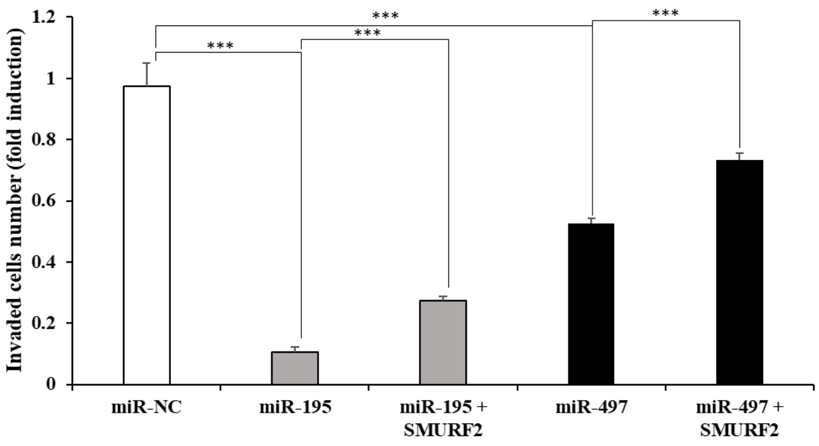

D

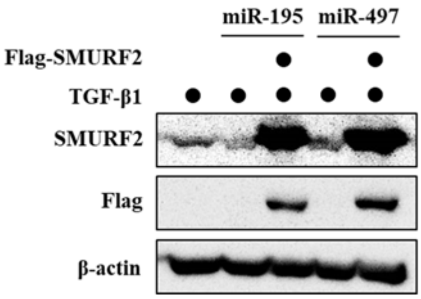

Fig. S8. The effects of Smurf2 on miR-195/497-mediated the reduction of cell proliferation. A549 cells were transfected with the mimics for miR-NC, miR-195, or miR-497 alone, or together with flag-SMURF2, and then treated with 1 ng/ml TGF-β1. (A) WST assay was performed to measure the cell viability. Two-tailed *t*-test was used for statistical analysis. The data represent mean ± SD; *P < 0.05, **P < 0.005, ***P < 0.001 (B, C) Matrigel invasion assay was used to check the invasive ability of the cells. The cells that invaded through the Matrigel were fixed and stained with giemsa (10× magnification). Scale bars indicate 100 µm. The number of invaded cells for each group was normalized to the control. The data are shown as the mean ± SD (*P < 0.05, **P < 0.005, ***P < 0.001, Student′s t-test). (D) Western blot analysis to detect SMURF2 protein levels

A

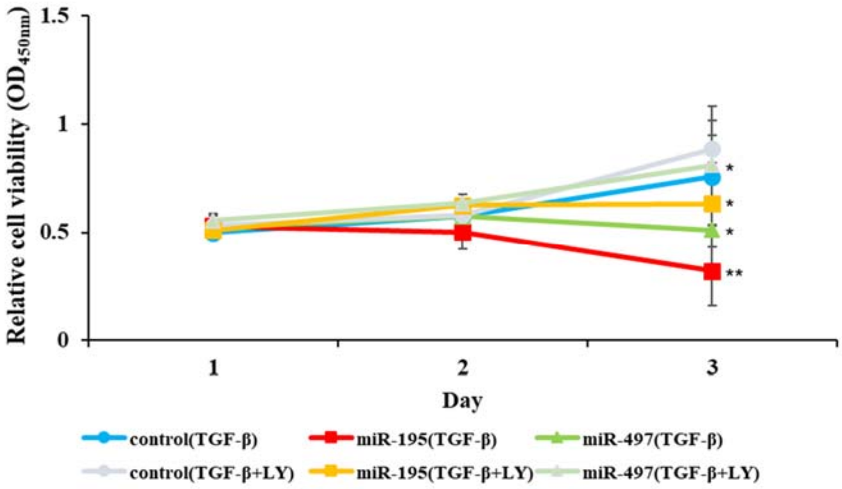

Relative cell viability (OD450nm)
1.5
1
0.5
0
1
2
3
Day
*
*
*
**
control(TGF-β)
miR-195(TGF-β)
miR-497(TGF-β)
control(TGF-β+LY)
miR-195(TGF-β+LY)
miR-497(TGF-β+LY)

B

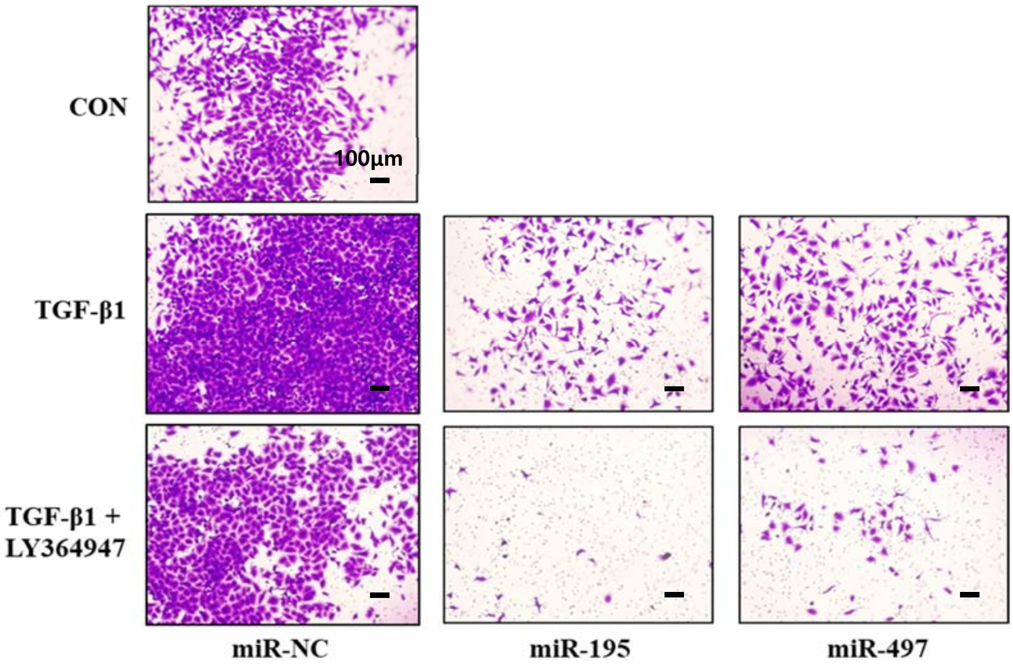

CON
100μm
TGF-β1
TGF-β1 +
LY364947
miR-NC
miR-195
miR-497

C

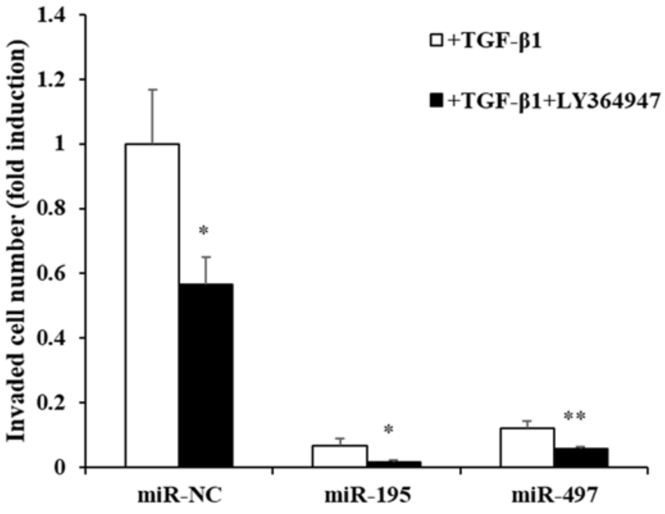

Fig. S9. The effects of miR-195 and miR-497 on cell viability and invasion in treatment with TGF-β1 and LY364947. A549 cells transfected with the mimics for miR-NC, miR-195 or miR-497 were treated with 1 ng/mL TGF-β1, and then treated with or without 100 ng/mL LY364947. (A) WST assay was performed to measure the cell viability. A two-tailed *t*-test was used for statistical analysis. The data represent mean ± SD; *P < 0.05, **P < 0.005, ***P < 0.001 (B, C) Matrigel invasion assay was used to check the invasive ability of the cells. The cells that invaded through the Matrigel were fixed and stained with giemsa (10× magnification). Scale bars indicate 100 μm. The number of invaded cells for each group was normalized to the control. The data are shown as the mean ± SD (*P < 0.05, **P < 0.005, ***P < 0.001, Student′s t-test).
